# Usefulness of Endoscopic Ultrasound with the Jelly-Filling Method for Esophageal Varices

**DOI:** 10.3390/diagnostics11091726

**Published:** 2021-09-20

**Authors:** Tsunetaka Kato, Takuto Hikichi, Jun Nakamura, Mika Takasumi, Minami Hashimoto, Ryoichiro Kobashi, Takumi Yanagita, Tadayuki Takagi, Rei Suzuki, Mitsuru Sugimoto, Yuki Sato, Hiroki Irie, Yoshinori Okubo, Masao Kobayakawa, Hiromasa Ohira

**Affiliations:** 1 Department of Endoscopy, Fukushima Medical University Hospital, Fukushima 960-1295, Japan; tsune-k@fmu.ac.jp (T.K.); junn7971@fmu.ac.jp (J.N.); mi-hashi@fmu.ac.jp (M.H.); rkobashi@fmu.ac.jp (R.K.); yoshi-o@fmu.ac.jp (Y.O.); mkobaya@fmu.ac.jp (M.K.); 2 Department of Gastroenterology, Fukushima Medical University School of Medicine, Fukushima 960-1295, Japan; paper@fmu.ac.jp (M.T.); takumi-y@fmu.ac.jp (T.Y.); daccho@fmu.ac.jp (T.T.); subaru@fmu.ac.jp (R.S.); kita335@fmu.ac.jp (M.S.); dorcus@fmu.ac.jp (Y.S.); hirokiri@fmu.ac.jp (H.I.); h-ohira@fmu.ac.jp (H.O.); 3 Medical Research Center, Fukushima Medical University, Fukushima 960-1295, Japan

**Keywords:** endoscopic ultrasound, endoscopy, esophageal varices, jelly, portal hypertension

## Abstract

Although the importance of endoscopic ultrasound (EUS) for esophageal varices (EVs) has been demonstrated, it is difficult to obtain sufficient EUS images with the water-filling method because of poor water stagnation in the esophagus. In this study on EVs, we aimed to evaluate the usefulness of the jelly-filling method for EUS. Consecutive patients who underwent EUS for EVs were included. The quality of EUS images, the diagnostic ability of the presence of blood vessels inside and outside the esophageal wall, and the procedure time were compared between the jelly-filling and water-filling methods. Thirty cases were analyzed (jelly-filling method in 13 and water-filling method in 17). The EUS image quality score was significantly higher in the jelly-filling method (jelly vs. water; three points vs. two points, *p* < 0.001). Additionally, EUS image quality scores in both nonexperts and experts were significantly higher in the jelly-filling method. The diagnostic ability of the presence of perforation veins was significantly higher in the jelly-filling method (jelly vs. water; 100% vs. 52.9%, *p* = 0.004). However, the procedure time was significantly longer in the jelly-filling method (*p* = 0.024). In conclusion, EUS using the jelly-filling method for EVs provided sufficient image quality.

## 1. Introduction

Esophageal varices (EV) are common in patients with portal hypertension such as liver cirrhosis [1]. EVs are formed as collateral blood vessels in portal hypertension, and bleeding arising from them has a fatal prognosis [2,3,4]. Hemostasis with endoscopic variceal ligation (EVL) has been reported to be useful in cases of EV bleeding [5,6,7]. Besides EVL, endoscopic injection sclerotherapy (EIS) [8,9,10,11,12] is used as a prophylactic treatment for EVs with bleeding risk. EIS is a popular treatment for EVs in Japan, and the evaluation of the EV site is important as a pretreatment examination, along with the evaluation of portal hemodynamics. The usefulness of endoscopic ultrasound (EUS) for the evaluation of hemodynamics in the EV site has been reported [13,14,15,16,17]. Regarding the EUS device, an ultrasound probe [13,14,15,16,17] and a specialized EUS scope are utilized [17,18,19]. The EUS method using an ultrasound probe is a simple method that can be followed by conventional endoscopic observation and is commonly performed by the water-filling method [13]. However, the water-filling method has several issues, including difficulty keeping water in the esophagus, artifacts in EUS images due to turbidity and bubbles in the injected water, and the risk of aspiration pneumonia due to regurgitation. Thus, obtaining sufficient EUS images for EVs is difficult.

Esaki et al. [20] and Hanaoka et al. [21] reported an EUS method using jelly instead of water for esophageal lesions. The jelly-filling method is based on the fact that jelly stays in the esophagus for a long time because of its high viscosity when compared with water. However, to the best of our knowledge, no previous method has shown the usefulness of EUS using the jelly-filling method for EV diagnosis. Thus, this study was conducted to evaluate the usefulness of EUS with the jelly-filling method for EVs.

## 2. Methods

### 2.1. Study Setting

Fukushima Medical University Hospital is one of the largest hospitals in Fukushima Prefecture in Japan. The indications for endoscopic treatment for EVs were elective cases with a history of bleeding and prophylactic cases with a high risk of bleeding. EVs with a high risk of bleeding were defined as those with a morphology of F2 or higher or a red sign on the basis of the classification by the Japan Society of Portal Hypertension [22]. In our institution, EUS with the water-filling method was performed until March 2019, whereas EUS with the jelly-filling method has been performed since April 2019.

**Figure 1:**
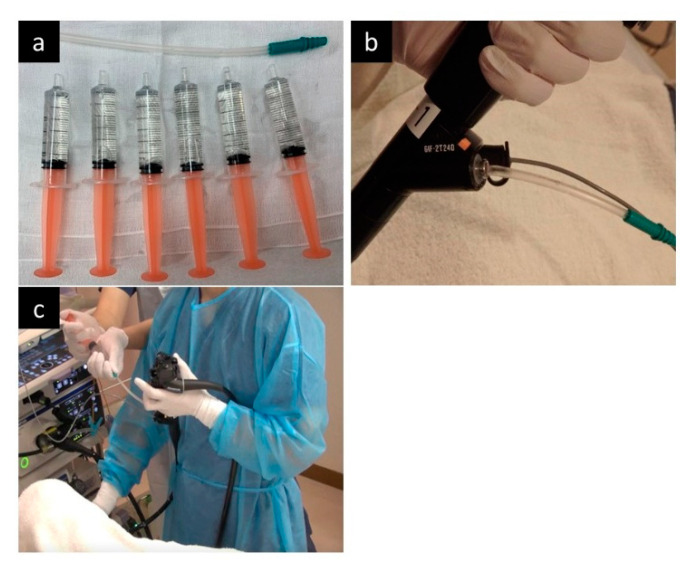
The jelly-filling method in the endoscopic ultrasonography (EUS). (**a**) Jellies is prepared in 5-mL syringes, and a short 14Fr catheter is also prepared. (**b**) After inserting the endoscope, insert the 14Fr catheter through the wide forceps channel and insert the ultrasound probe through the other forceps channel. (**c**) During EUS observation, additional jelly injections are performed by the assistant, not the operator.

EUS was performed under sedation with midazolam, but without antispastic agents. The jelly-filling method was performed using a two-channel endoscope (GIF-2T240; Olympus, Tokyo, Japan) according to the method of Hanaoka et al. [21] (Figure 1). After confirming the presence of EVs, a 20 MHz ultrasound probe (UM-3R; Olympus, Tokyo, Japan) was inserted through the left forceps channel (channel diameter: 2.8 mm), and a 14-Fr catheter was inserted through the right forceps channel (channel diameter: 3.7 mm). Next, approximately 20 mL of water-soluble lubricating jelly (Through Projelly; Kaigen Pharma Corp., Osaka, Japan) was injected into the 14-Fr catheter into the esophagus, and observation by EUS was started. During EUS observation, jelly was added as necessary. The jelly is used as a lubricant for the surface of the endoscope during the endoscopic procedure. The ingredients of the jelly are water, propylene glycol, glycerin, hydroxyethyl cellulose, paraben, and carbomer. After EUS observation, the jelly was aspirated as much as possible.

On the other hand, the water-filling method was performed using a one channel endoscope (GIF-Q260J or GIF-H290Z; Olympus Medical Systems Corp., Tokyo, Japan) and an ultrasound probe. After filling the stomach with approximately 300 mL water, the esophageal lumen was filled with water, and EUS observation was performed while adding water using the forward water supply function.

The study was conducted according to the guidelines of the Declaration of Helsinki, and it was approved by the Institutional Ethics Committee of Fukushima Medical University (protocol code: 2406, date of approval: 18 February 2021).

### 2.2. Study Design and Patients

We retrospectively collected and analyzed clinical data and stored EUS images (not video) from electronic medical records and endoscopic databases at Fukushima Medical University Hospital. Thirty consecutive patients with EVs who underwent EUS before endoscopic treatment from April 2017 to March 2020 were included in this study. In addition, subgroup analyses were conducted based on the operator’s expertise.

### 2.3. Outcomes and Definitions

The primary outcome was the quality of EUS images, and the secondary outcomes were the diagnostic ability of the presence of blood vessels inside and outside the esophageal wall, procedure time, and adverse events (AEs). The quality of EUS images and procedure time of experts and nonexperts were also subanalyzed. Operators were defined as “experts” if they had more than 10 years of endoscopy experience, and “nonexperts” if they had less than 10 years of endoscopy experience. Nonexperts were physicians who had insufficient experience in EUS for EVs but were able to perform EUS for gastric cancer. Each outcome was compared between the jelly-filling method and the water-filling method.

**Figure 2:**
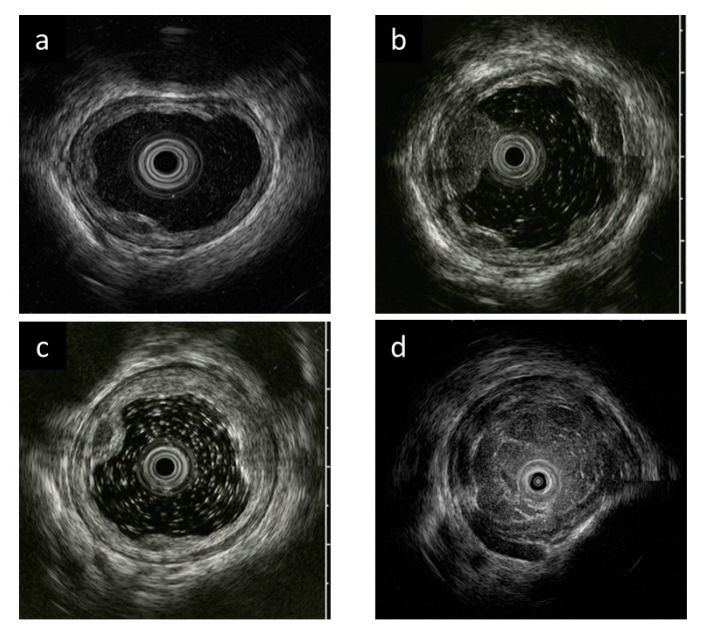
Evaluation criteria for endoscopic ultrasonography (EUS) image quality. (**a**) Excellent (4 points). Clear images with no debris-like artifacts in EUS. (**b**) Good (3 points). A few debris-like artifacts are present, but do not interfere with the evaluation of esophageal varices (EVs) in EUS. (**c**) Moderate (2 points). Debris-like artifacts are noticeable, but do not interfere with EV evaluation in EUS. (**d**) Poor (1 point). Difficult to assess EV due to debris-like artifacts and poor esophageal lumen expansion in EUS.

The quality of EUS images and the ability to diagnose the presence of vessels inside and outside the esophageal wall were evaluated by discussion between two experts (T.K. and T.H.). The quality of EUS images was assessed for each case using a 4-point score (4 points: excellent, 3 points: good, 2 points: moderate, and 1 point: poor) (Figure 2), and the median of all EUS image scores of the case was defined as the image quality score for that case.

Regarding the vessels inside and outside the esophageal wall [22], peri-esophageal vein (Peri-v), para-esophageal vein (Para-v), and perforating vein (Pv) were evaluated; “clear” was defined when the presence or absence of these vessels could be clearly assessed from the stored images and examination report, and “unclear” was defined when the presence or absence of these vessels was difficult to determine. The procedure time was defined as the time from the first EUS image to the last EUS image.

### 2.4. Statistical Analysis

Values are reported as the medians with ranges. Statistically significant differences between patient characteristics and EUS results were assessed using Mann–Whitney U test for continuous variables, and chi-square test and Fisher’s exact test for discrete variables. Differences were considered to be significant at *p* < 0.05. This analysis was performed using the SPSS software (Version 21 for Windows; IBM Corp, Armonk, NY, USA).

## 3. Results

### 3.1. Patient Characteristics

Thirty patients (13 with the jelly-filling method and 17 with the water-filling method) were identified. Table 1 shows the background of the patients. All patients had cirrhosis, and the indications for endoscopic treatment of EV were prophylactic in all cases for the jelly-filling method, and prophylactic in 13 cases and elective in four cases for the water-filling method. There were no significant differences in age, sex, cause of liver cirrhosis, indication for endoscopic treatment, or endoscopic findings of EV between the two groups. There was also no difference in the experience of operators between the two groups (Table 2).

### 3.2. EUS Image Quality

**Figure 3:**
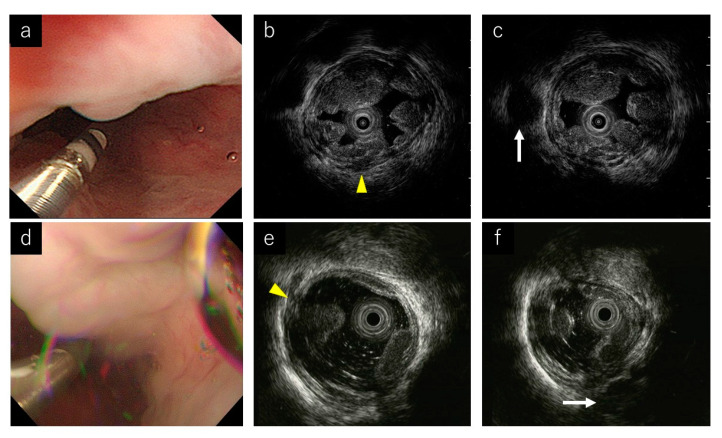
Endoscopic ultrasound (EUS) images of representative cases. (**a**) Endoscopic image during the jelly-filling method. (**b**,**c**) EUS images of the jelly-filling method. Yellow arrow head shows a perforating vein (Pv). White arrow shows a para-esophageal vein (Para-v). (**d**) Endoscopic image during the water-filling method. (**e**,**f**) EUS images of the water-filling method. Yellow arrow head shows a perforating vein (Pv). White arrow shows a para-esophageal vein (Para-v).

The EUS image quality score was significantly higher in the jelly-filling method (jelly vs. water; 3 points vs. 2 points, *p* < 0.001) (Table 2, Figure 3). Additionally, the score was significantly higher in the jelly-filling method for both experts (jelly vs. water; 3 points vs. 2 points, *p* = 0.007) and nonexperts (jelly vs. water; 3 points vs. 2 points, *p* = 0.003) (Table 3). There was no significant difference in the diagnostic ability of the presence of Peri-v and Para-v between the two methods, but that of Pv was significantly higher in the jelly-filling method (jelly vs. water; 100% vs. 52.9%, *p* = 0.004) (Table 2). The median procedure time was significantly longer in the jelly-filling method (jelly vs. water; 6 min vs. 4 min, *p* = 0.024) (Table 2).

### 3.3. Procedure Time and AEs

The median procedure time was significantly longer in the jelly-filling method (jelly vs. water; 6 min vs. 4 min, *p* = 0.024) (Table 2). In the subanalysis, there was no significant difference in procedure time between the two methods for the experts, but the procedure time was significantly longer for nonexperts using the jelly-filling method (jelly vs. water; 8 min vs. 5 min, *p* = 0.036) (Table 3).

In both methods, no AEs such as aspiration pneumonia were observed during or after EUS (Table 2).

## 4. Discussion

In this study, we showed that the jelly-filling method provided better EUS images for EVs than the water-filling method. Additionally, the jelly-filling method did not cause AEs, demonstrating that it can be performed safely. To the best of our knowledge, this is the first report to demonstrate the usefulness of the jelly-filling method of EUS for EVs.

In EUS using an ultrasound probe, it is necessary to fill the esophageal lumen with a liquid such as water. However, in the water-filling method, the water injected into the esophagus flows quickly into the stomach due to gravity, and air in the stomach flows back into the esophagus. Consequently, the water mixes with mucus in the esophagus and stomach, resulting in insufficient EUS images or debris-like artifacts in the EUS images. Additionally, water can cause aspiration if it flows back into the mouth [23]. To solve these issues of the water-filling method, an EUS method involving the placement of water and an ultrasound probe in a condom, instead of directly injecting water into the esophagus, has been reported [24]. However, the condom method was problematic because the procedure was complicated and the condom was in contact with the lesion, which sometimes affected the accuracy of the evaluation. Additionally, the use of a condom may not prevent the reflux of air from the stomach into the esophagus.

Then, EUS using the jelly-filling method has also been reported as a new method for EUS [20,21]. Because jelly is more viscous than water, it can be easily stored in the narrow lumen of the esophagus, where it can remain for a long time. It also has the advantage of being less susceptible to reflux of air from the stomach. Esaki et al. [20] showed that the jelly-filling method demonstrated significantly higher diagnostic performance than the water-filling method in diagnosing the depth of cancer in superficial esophageal cancer. Moreover, Hanaoka et al. [21] reported a different jelly-filling method using a two-channel scope as a way to obtain more stable EUS images. By using a two-channel scope, the ultrasound probe can be inserted through one channel and jelly can be injected into the other channel. This allows for the continuous injection of jelly when it is flowing into the stomach, and consequently, high-quality EUS images with less air and debris can be obtained. In this study, we introduced the jelly-filling method of EUS using a two-channel scope. Our results showed that significantly better EUS images were obtained with the jelly-filling method for not only experts, but also nonexperts.

Regarding the diagnostic ability of the presence of blood vessels inside and outside the esophageal wall, that of Pv was significantly higher in the jelly-filling method. Pv is a vessel that connects the Para-v to the EV. It is difficult to evaluate the presence of Pv when artifacts are prominent in the esophagus by the water-filling method. However, the jelly-filling method made an evaluation of the presence of Pv easier than the water-filling method because expansion of the esophageal lumen could be maintained for Pv evaluation. The jelly-filling method of EUS is useful in selecting the appropriate treatment for EVs because EIS is preferable to EVL in patients with Pv on EUS [25,26,27]. In patients with Pv, EVL has a high recurrence rate, so EIS is the preferred treatment. On the other hand, if the Pv is thicker than 5 mm, the sclerosing agent may escape into the systemic circulation during EIS, and there is a risk of AEs such as embolism. Therefore, it is necessary todevise a technique such as using ethanol injection even during EIS [12]. Sufficient EUS image quality and accurate determination of the presence or absence of Pv are important not only for therapeutic efficacy but also for reducing the occurrence of AEs. As Peri-v and Para-v are outside the esophageal wall, they can be evaluated independently of artifacts in the esophagus. Therefore, there were no significant differences between the two groups with Peri-v and Para-v.

Regarding the procedure time, the jelly-filling method was longer. This may be because the evaluation of EV and EV-related vessels by the water-filling method was difficult because of the outflow of water into the stomach, and the examination was unavoidably completed over a short period of time in some cases. However, in the jelly-filling method, EUS examination could be concentrated on, and many cases were observed with certainty over a long period of time. Indeed, the longer procedure time in nonexperts with less EV experience reflected this fact. The longer procedure time in the jelly-filling method does not depend on the jelly-filling method itself but only on the operator. However, to describe the usefulness of the jelly-filling method from the perspective of procedure time, we feel that it is important to educate nonexperts to accurately judge the items to be evaluated in a short time.

The learning curve was not taken into account in this study. However, all the operators who performed the jelly-filling method had experiences of the water-filling method. In addition, the jelly-filling method can be simplified using a two-channel scope. In fact, even a nonexpert operator can easily learn the expert technique by observing one case. Moreover, at our institution, experienced medical staff play the role of assistants for EUS. Therefore, an operator who has experience with the water-filling method can perform the jelly-filling method from the first case without any problem.

Recently, the gel immersion method has been reported in Japan as an EUS technique with a similar concept to the jelly-filling method [28,29,30]. GIE has developed to improve the field of view of the endoscope by using a viscous gel product and was originally developed to secure the field of view during hemostasis in gastrointestinal bleeding [31]. We have just started the clinical application of gel immersion method, and the difference between the jelly-filling method and gel immersion method in EUS for EV will be discussed in the future. In our experience of performing EUS with the gel immersion method, the gel flowed more easily into the stomach and the stagnation time in the esophagus was shorter in the gel immersion method than in the jelly-filling method.

Our study has several limitations. First, this is a single-center study with a small number of patients. Second, this study is not a randomized control trial, and the physicians who evaluated the EUS images were not blind. Additionally, different scopes were used between the two groups. Although the water-filling method used a one-channel scope, the jelly-filling method used a two-channel scope. In the water-filling method, additional water can be injected using the water jet function of the scope or by injecting water through the forceps channel while the ultrasound probe is inserted into the scope. In contrast, when the jelly-filling method is performed using a one-channel scope, additional injection of jelly cannot be performed while the ultrasound probe is inserted into the scope because of the high viscosity of jelly. This is why we used a two-channel scope for the jelly-filling method. However, the use of a two-channel scope in the jelly-filling method is an important aspect of the technique. Third, the EUS images and procedure time were retrospectively evaluated from the stored images and electronic medical records. However, no obvious bias was found in these data because the timing and procedure of EUS images were standardized in our institution. Fourth, we did not compare the actual presence of Peri-v, Para-v, and Pv with varicealography during EIS and computed tomography. Fifth, EUS was not performed by a specific operator but by multiple operators. However, we could evaluate the quality of EUS images and procedure time in nonexpert operators.

In conclusion, for EVs, EUS using the jelly-filling method was superior to EUS using the water-filling method in that it provided better EUS images, even in nonexpert operators, and was also excellent in evaluating Pv. However, further studies with a large number of patients are needed.

## Figures and Tables

**Table 1:** Comparison of patient background between the jelly-filling method and the water-filling method.

Evaluated Items	Jelly-Filling Method (*n* = 13)	Water-Filling Method (*n* = 17)	*p*-Value
Age (years), median (range)	66 (40–77)	66 (43–83)	0.630
Sex, *n*			0.440
Male	10	10	
Female	3	7	
Cause of liver cirrhosis, *n*			
Alcoholic/PBC/NASH/virus/unknown	5/3/3/1/1	6/4/2/3/2	0.205
Indication of endoscopic treatment, *n*			
Elective/prophylactic	0/13	4/13	0.113
Endoscopic findings of esophageal varices, *n*			
Location			
Li/Lm/Ls	4/7/2	1/12/4	0.332
Form			
F1/F2/F3	1/12/0	2/15/0	0.717
Color			
Cb/Cw	13/0	16/1	0.381
Red color sign (RC)			
RC0/≥RC1	4/9	10/7	0.133

Location: Li: Locus inferior, Lm: Locus medialis, Ls: Locus superior. Form: F1; Straight, small-caliber varices, F2: Moderately enlarged, beady varices, F3: Markedly enlarged, nodular or tumor-shaped varices. Color: Cb; Blue varices, Cw; White varices. Red color sign: RC0: Absent, RC1: Small and localized red color findings.

**Table 2:** Comparison of the results of endoscopic ultrasonography (EUS) observation between the jelly-filling method and the water-filling method.

Evaluated Items	Jelly-Filling Method (*n* = 13)	Water-Filling Method (*n* = 17)	*p*-Value
EUS operator			1.000
Nonexpert	6	8	
Expert	7	9	
EUS image quality (points) *	3 (3–4)	2 (1–4)	<0.001
Procedure time (min) *	6 (3–10)	4 (2–10)	0.024
Observation of Peri-v			0.238
Clear	13	14	
Unclear	0	3	
Observation of Para-v			0.053
Clear	13	12	
Unclear	0	5	
Observation of Pv			0.004
Clear	13	9	
Unclear	0	8	
Adverse events	0	0	–

* Data are shown as the median (range). Peri-v: Peri-esophageal vein, Para-v: Para-esophageal vein, Pv: Perforating vein.

**Table 3:** Sub-analysis of EUS image quality and procedure time according to the operator’s experience with EUS procedures.

Evaluated Items	Jelly-Filling Method (*n* = 13)	Water-Filling Method (*n* = 17)	*p*-Value
Expert, *n*	7	9	
EUS image quality (points)	3 (3–4)	2 (2–3)	0.007
Procedure time (min)	5 (3–7)	4 (2–7)	0.329
Nonexpert, *n*	6	8	
EUS image quality (points)	3 (3–4)	2 (1–3)	0.003
Procedure time (min)	8 (6–10)	4 (3–10)	0.036

Data are shown as the median (range).

## Data Availability

Data are available on request due to restrictions, e.g., privacy or ethical.
